# Identification of a minimal strong translation enhancer within the 5′-untranslated region of *OsMac3* mRNA

**DOI:** 10.5511/plantbiotechnology.24.0909a

**Published:** 2024-12-25

**Authors:** Hiromi Aoki-Mutsuro, Ryoko Tamukai, Miho Fukui, Mai Wajiki, Tomohiro Imamura, Lyubov A. Ryabova, Mikhail V. Schepetilnikov, Hiroshi Teramura, Hiroaki Kusano, Hiroaki Shimada

**Affiliations:** 1 Department of Biological Science and Technology, Tokyo University of Science, Katsushika, Tokyo 125-8585, Japan; 2 Institut de Biologie Moléculaire des Plantes, CNRS UPR2357, 67084 Strasbourg, France

**Keywords:** mRNA, non-coding RNA, protein production, stem and loop structure, translation

## Abstract

The long 5′ untranslated region (5′UTR) exhibits enhancer activity in translation of rice *OsMac3* mRNA. In this report, we describe elements of *OsMac3* 5′UTR that may be responsible for its enhancer activity, including a long uORF and several secondary structure elements. *OsMac3* 5′UTR can be dissected into three stem-loop structures SL1, small SL and SL2, where the uORF starts within SL1 and ends within SL2. As expected, uORF inhibits translation of downstream ORF since deletion of the uORF AUG or the SL1 stem-loop increases translation by approximately two-fold. Thus, the 158 nt 3′ region of the 5′UTR lacking SL1 together with the AUG uORF, which has significant enhancer activity, was named dMac3. We investigated two critical regions within dMac3 mRNA that influence its translation: SL2, which destabilization potentially decreases translation activity, and another 13 nt located downstream of SL2. We further confirmed that dMac3 promotes mRNA translation initiation in an in vitro translation system and during transient expression in either cultured cells or *Nicotiana benthamiana* leaves. Thus, the dMac3 5′UTR is a useful tool for efficient protein production in various in vitro and in vivo translation systems.

## Introduction

The rice *OsMac* family genes, *OsMac1*, *OsMac2* and *OsMac3*, contain long 5′ untranslated regions (5′UTRs). The UTRs of these genes exhibit strong translational activity and thus enhance the translation of downstream open reading frames (ORFs) (Aoki et al. 2014; Teramura et al. 2012). The 5′UTR of the *OsMac1* mRNA forms a complex secondary structure, which is important for its enhancer activity (Mutsuro-Aoki et al. 2021).

The 5′UTRs in the *OsMac2* and *OsMac3* mRNAs are also predicted to form stem-loop structures. However, their predicted structures differ significantly from that of *OsMac1* and reveals only slight similarity in the nucleotide sequences of their 5′UTRs (Aoki et al. 2014). Therefore, it is unclear which 5′UTR elements are responsible for their positive effects on translation.

A scanning model for translation initiation of eukaryotic mRNA was proposed by Kozak (1999). The initiation step of translation involves a large number of eukaryotic translation initiation factors (eIFs) that are responsible for cap-dependent translation initiation, including the initial binding of the 43S preinitiation complex (43S PIC) to the capped 5′end of the mRNA. The 43S PIC scans the mRNA 5′UTR until it recognizes the first AUG initiation codon. At this point, the 60S ribosomal subunit joins the complex to start the elongation step of protein synthesis at the downstream ORF.

The presence of a strong secondary structure and specific elements within the 5′UTR can impede ribosome scanning and thus regulate translation efficiency (Hinnebusch et al. 2016; Kozak 1988, 2007; Leppek et al. 2018). Increasing number of reports have demonstrated a role of short upstream open reading frames (uORFs) in mRNA translational control (Calvo et al. 2009; Hinnebusch et al. 2016; Schepetilnikov and Ryabova 2018). The long uORF or/and combination of several uORFs are considered to be main translation inhibitors, where their translation normally downregulates translation of the downstream ORF via the process called reinitiation (Calvo et al. 2009; Kozak 2001; Luukkonen et al. 1995; Schepetilnikov et al. 2013). Efficiency of reinitiation also depends on different physiological conditions and increases upon activation of the target of rapamycin protein kinase (TOR; Schepetilnikov et al. 2013, 2017). To overcome the inhibitory effect of short uORFs and strong secondary structures, a mechanism of ribosomal shunting has been proposed that allows ribosomes to bypass these inhibitory elements (Ryabova et al. 2006). In the ribosomal shunt model, formation of the leader hairpin structure triggers shunting by bringing the shunt landing site upstream of the main downstream ORF into proximity with a shunt take-off site downstream of short uORF. In this model, translation and termination of short uORF is a prerequisite for reinitiation at downstream main ORF (Ryabova and Hohn 2000).

Viral mRNAs often contain 5′UTRs carrying the internal ribosome entry sites (IRESs) which locates immediately upstream of the ORF. IRES functions as a direct binding site for the ribosome and allow polycistronic translation if located upstream of multiple ORFs within polycistronic viral mRNAs (Fernandez et al. 2002; Shababi et al. 2006; Vagner et al. 2001). The sequences showing the IRES functions have been found in some eukaryotic mRNAs such as maize *Hsp101* and *Adh1* genes. Ribosome binding to IRES is not dependent on cap-structure of mRNA (Jiménez-González et al. 2014; Mardanova et al. 2008). A unique case of polycistronic translation via reinitiation was found operating within the CaMV 35S RNA under control of viral protein TAV (translation transactivator/ viroplasmin) and TOR (Pooggin and Ryabova 2018; Schepetilnikov et al. 2011).

The 5′UTR of *OsMac3* is known to increase the translational efficiency of the downstream ORFs by 10- to 30-fold (Aoki et al. 2014). In this study, to elucidate the mechanism by which the 5′UTR of the *OsMac3* promotes translation of the downstream ORF, we constructed the series of 5′UTR mutants to determine the core region involved in translation enhancement. In this report, we discovered that the 3′ region exhibits high translational initiation activity in different in vitro and in vivo cellular background.

## Materials and methods

### Construction of reporter genes

The *GUS* gene was used as a reporter, which was PCR-amplified from pBI221 (Jefferson et al. 1987) and inserted into pENTR™/D-TOPO (Invitrogen, Carlsbad, CA, USA) to generate pENTR-35S-GUS (35S-GUS). The 5′UTR region in the *OsMac3* cDNA was inserted preceding the *GUS* gene in pENTR-35S-GUS to generate pENTR-35S-5′UTR-GUS (35S-5′UTR-GUS). Internal deletion, insertion, or substitution mutations were introduced into the 5′UTR using the megaprimer method (Ling and Robinson 1997) using specific primer sets that were designed to be mutagenized. Alternatively, fragments corresponding to some of mutant 5′UTRs were chemically synthesized. Resultant fragments containing mutant 5′UTRs were inserted preceding the *GUS* gene in pENTR-35S-GUS. CaMV ORFVII has shown strong inhibition of translation of the GUS ORF following ORFVII contained in a bicistronic mRNA (Mutsuro-Aoki et al. 2021). The fragment for the entire ORFVII (acc. no. M94887.1) was chemically synthesized and inserted into the region immediately upstream of 5′UTR in pENTR-35S-5′UTR-GUS and its derivatives. The 2.5-kb fragment of the *SPK* promoter (acc. no. E14792) was PCR-amplified using the plasmid containing the *SPK* gene, which is weakly expressed in rice cultured cells (Asano et al. 2002; Kawasaki et al. 1999). The 0.9-kb fragment of the *Ubiquitin* promoter (*RUBQ2*, acc. no. AF484682) was taken by PCR-amplification using rice genomic DNA. These promoter fragments were replaced to the *35S* promoter in pENTR-35S-5′UTR-GUS. The DNA fragment for the *SPK* promoter was replaced with the fragment of the *35S* promoter in pENTR-35S-GUS to generate pSPK-GUS. The DNA fragment of dMac3 was introduced into the pSPK-GUS to create pSPK-dMac3-GUS. The fragment of *RUBQ2* promoter was introduced into pENTR-35S-GUS by replacement with the *35S* promoter to generate pRUBQ2-GUS. pRUBQ2-dMac3-GUS was constructed by inserting the dMac3 fragment into pRUBQ2-GUS.

3xFLAG-tagged DsRED2 protein was used for in vitro protein synthesis and transient gene expression in *Nicotiana benthamiana*. For preparation of the 3xFLAG-tagged protein, the fragment encoding DsRED2 was PCR-amplified from pDsRED2 (Takara Bio Inc., Kusatsu, Japan) and connected with the chemically synthesized fragment, 5′-ACGCGTGATTACAAGGATCATGATGGCGATAAGGATCATGATATCGATTACAAGGATGATGATGATAAGTAG, for 3xFLAG tag at their 3′ ends. The resultant fragment was inserted into pEU-dMac3 (Suzuki et al. 2020), which was constructed by replacing the E01 sequence with the dMac3 region, a portion of the 5′ untranslated region of OsMac3 mRNA, in pEU-E01 (CellFree Sciences Co., Ltd., Ehime, Japan) to generate a plasmid pdMac3-DsRED2-FLAG (dMac3-DsRED2-FLAG). In parallel, the fragment of 3xFLAG-tagged DsRED2 was cloned in pEU-E01, in which the region for E01 sequence had been removed, to generate a plasmid pDsRED2-FLAG (DsRED2-FLAG). To express in the transformants, *35S* promoter was introduced upstream of the reporter genes. Binary plasmids were produced using pGWB1 (Nakagawa et al. 2007) via LR clonase reaction.

### Determination of translational efficiency using rice protoplasts

Translational efficiency was determined using protoplast of the cultured cells as described previously (Mutsuro-Aoki et al. 2021). Cultured rice (*Oryza sativa* L.) *Oc* cells were suspension-cultured in Murashige and Skoog medium (Murashige and Skoog 1962) and used for the reporter analysis. The *Oc* cell line was supplied by RIKEN Bioresource Center, Japan. Rice protoplast cells were prepared from cultured cells. They were transformed using DNA of the desired reporter gene by the PEG method (Yoo et al. 2007). The cells were incubated for 16 h at 26°C in WI buffer (4 mM MES (pH 5.7), 0.5 M mannitol, and 20 mM KCl) and then harvested by centrifugation. The resultant protoplast cells were suspended in GUS extraction buffer (0.5 mM Tris-HCl (pH 7.0), 10 mM EDTA, 1% TritonX-100, and 1% Nonidet P-40 (Sigma-Aldrich, St. Louis, MO, USA)). The aliquots were immediately taken for *GUS* reporter gene assays using 4-methylumbelliferyl-β-D-glucuronide (Nacalai Tesque Inc., Kyoto Japan). GUS activity was measured by the fluorometric assay method (Pooggin et al. 2000). Relative GUS activity was estimated as the relative value of GUS activity relative to the level of the reporter mRNA, which was determined using real-time quantitative RT-PCR. Due to the values that varied depending on the viability of the cells used in each experiment, the relative GUS activities were adjusted based on the value of the observed GUS activity of dMac3 in a simultaneous experiment. Relative translational efficiency of each reporter construct was determined as the relative value of the observed GUS activity to that of the reporter gene containing dMac3. For determining the relative GUS activities, values of cell factor were taken by the results of qRT-PCR that were multiplied by the cell factors depending on the cell viability whose values were obtained by the GUS activity on the dMac3 reporter gene. The data were analyzed statistically using Dunett’s test or Student’s *t*-test. In parallel, *35S-GFP* (Aoki et al. 2014) was introduced into protoplast cells. The amount of fluorescence signals derived from the introduced GFP gene was measured and used to monitor the efficiency of translation in protoplast cells.

### Protein translation using in vitro translation system and detection of the produced DsRED2

mRNAs were synthesized using an in vitro transcription system. They were subjected to preparation of the corresponding proteins using a wheat germ protein synthesis kit (CellFree Science, Yokohama, Japan). The general procedure followed the manufacture’s protocol. The generated proteins were evaluated by SDS-polyacrylamide gel electrophoresis (PAGE) and protein blot analysis. The 3xFLAG-tagged DsRED2 protein was detected using the anti-FLAG M2 antibody (Sigma-Aldrich).

Fluorescence emission by DsRED2 was detected using Image Quant LAS4010 (GE Healthcare, Chalfont Saint Giles, UK) as follows. The light source: cy3 Green (EpiRGB, 520 nm), the fluorescence detection filter: 575DF20, the lens: F 0.85, exposure time: 1 s. The visible images were taken under the epi-illumination condition with 1/30 s exposure. The captured images were analyzed using Image Quant TL software (GE Healthcare). The amount of protein was estimated based on the density of the band.

### Generation of rice transformants and transient expression of the introduced gene

The binary plasmids were introduced into rice callus by the *Agrobacterium*-mediated method (Hiei et al. 1994). The hygromycin-resistant transformants were selected on Murashige–Skoog plates (Murashige and Skoog 1962) supplemented with 50 mg l^−1^ hygromycin B (Fuji-Film Wako Pure Chemical Corp., Tokyo, Japan). Transformant callus was cultured on Murashige–Skoog plates under a long-day condition. The generated DsRED2 was detected by protein blot analysis and by the fluorescence emission procedure as described above.

### Transient expression in tobacco leaves

Introduction of the plasmids into tobacco (*Nicotiana benthamiana*) leaves was performed by the agroinfiltration method (Liu and Lomonossoff 2002). Tobacco was grown at 22°C under a 12 h light/12 h dark condition. *Agrobacterium tumefaciens* cells transformed by the appropriate binary plasmid were cultured in YEB medium (0.5% beef extract, 0.1% Bacto peptone, 0.1A% yeast extract, 0.5% sucrose) containing 50 µg l^−1^ kanamycin at 28°C for 2 days. Bacterial broth was adjusted to OD=1.0 by addition of YEB medium. This was further diluted 3 times by YEB medium immediately before the operation of infection. The bacterial solution was injected into the backside of a young tobacco leaf using a small syringe. Plants infected with *Agrobacterium* were incubated at 22°C under a 12 h light/12 h dark condition for 5 days.

### Realtime quantitative RT-PCR analysis

Total RNA was prepared from each tissue as described previously (Imamura et al. 2007). First-strand cDNA was synthesized from 1 µg of total RNA using a ReverTra Ace cDNA synthesis kit (Toyobo Co., Ltd., Osaka, Japan) with an oligo-dT (20) primer. Real-time quantitative PCR was performed as described previously (She et al. 2010) using Thunderbird SYBR qPCR mix (Toyobo). The levels of the reporter *GUS* transcripts, and *Actin1* (acc. no. AK100267) transcript were monitored by pairs of gene-specific primers: 5′-GCCGATGCAGATATTCGTA-3′ and 5′-CCATCACTTCCTGATTATTGA-3′, and 5′-CCCTCCTGAAAGGAAGTACAGTGT-3′ and 5′-GTCCGAAGAATTAGAAGCATTTCC-3′, respectively.

## Results

### The effect of stem-loop structures and a single uORF within the *OsMac3* 5′UTR on translation of the downstream ORF

**Figure 1:**
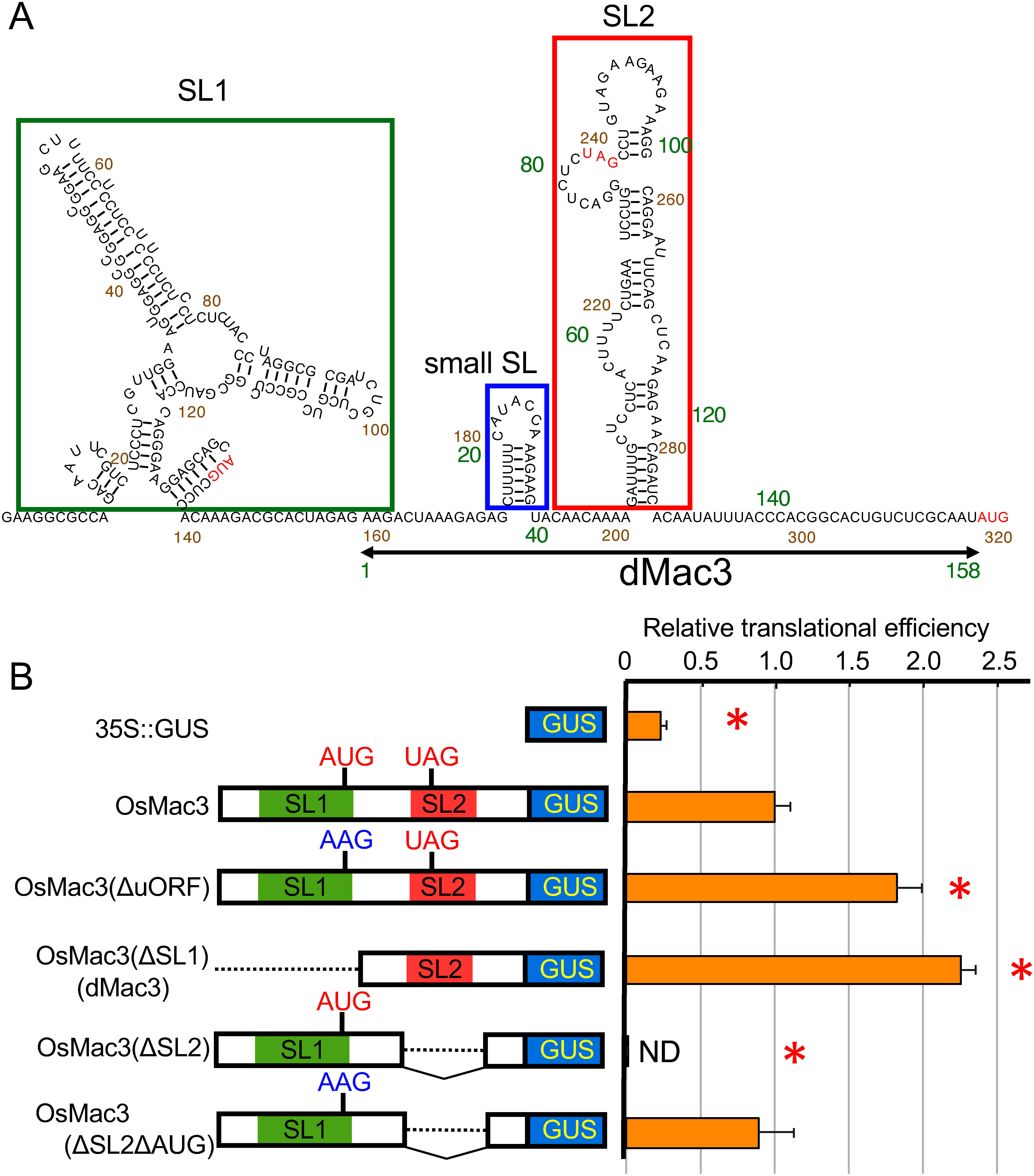
Figure 1. Characteristics of the 5′UTR in *OsMac3* mRNA and dMac3. (A) Predicted structure of the *OsMac3* 5′UTR and dMac3. Nucleotide sequence of the 5′UTR is shown. Local secondary structures in 5′UTR were predicted using the CentroidFold program (http://rtools.cbrc.jp/centroidfold). Initiation and termination codons on an uORF and the initiation codon of the downstream ORF are indicated by red letters. Regions for SL1, SL2 and small SL are boxed. Nucleotide numbers in OsMac3 5′UTR and dMac3 are indicated by orange and green letters, respectively. The region for dMac3 is indicated by an arrow. (B) Reporter assay on the translation efficiency in the modified 5′UTRs. Left panel indicates schematic representation of the reporter genes. OsMac3(ΔuORF), OsMac3(ΔSL1), OsMac3(ΔSL2) and OsMac3(ΔSL2ΔAUG) contain the nucleotide substitution in AUG of the uORF to AAG, deletion in the region of SL1, deletion in the region of SL2 and the nucleotide substitution in AUG of the uORF to AAG, and deletion in the region of SL2, respectively. OsMac3(ΔSL1) corresponds to the reporter gene containing dMac3. They were expressed by the *35S* promoter. The relative GUS activities were normalized against the *GUS* mRNA whose amount was estimated by real-time qRT–PCR. The results represent the means of three independent experiments. Error bars indicate the SD (*n*=3). The value of the relative translational efficiency with OsMac3 is set as 1.0. ND: not detected. Asterisks indicate significant differences in the translational efficiency of OsMac3 at *p*<0.01. The left panel indicate the schematic representation of the structures whose nucleotide sequences are shown in Supplementary Figure S1. The raw data of the GUS activity, relative GUS activity, and relative translational efficiency are shown in Supplementary Table S1.

The *OsMac3 5′UTR* secondary structure predicted by CentroidFold software contains two large stem-loops, SL1 and SL2, and a small one, small SL, located upstream of SL2 (Figure 1A). The uORF, encoding a 34 amino acid polypeptide, starts at the end of SL1 and ends in the middle portion of SL2. AUG of uORF is in a favorable initiation context (**A**GC **AUG** C) and contains A at position −3 with respect of the first nucleotide (nt) of the start codon (Supplementary Figure S1) (Kozak 1986). Thus, this uORF will be translated and interfere with small SL and SL2 secondary structure formation and can inhibit reinitiation of downstream ORF (Figure 1A).

To study the role of the *OsMac3* leader in translation initiation, we used a vector, where the *OsMac3* 5′UTR or its mutant derivatives were introduced upstream of the *GUS* gene under the *35S* promoter (Figure 1B). Translation enhancement activity of *OsMac3* 5′UTR and its derivatives was compared in transient expression system in rice protoplasts, followed by analysis of GUS activity.

A construct lacking uORF, OsMac3(ΔuORF), where the uORF AUG codon was mutated to AAG, showed more efficient expression of *GUS* ORF (1.8-fold), suggesting that uORF does indeed inhibit the translation reinitiation of the downstream ORF.

The Mutant OsMac3(ΔSL2) lacking the region of SL2 retained the initiation codon of the uORF in *OsMac3* by which a translation would occur from this AUG to generate the protein corresponding to that derived from the different frame in the *GUS* gene. Deletion of the region containing SL2 blocks *GUS ORF* translation reinitiation (Figure 1B, OsMac3(ΔSL2)), because that is not surprising giving that SL2 deletion results in the appearance of a longer uORF (121 nt) that partially overlaps with the *GUS* ORF and thus, prevents translation reinitiation of the downstream *GUS* ORF. Accordingly, substitution of AUG by AAG codon can fully restore GUS activity (Figure 1B, OsMac3(ΔSL2ΔAUG)). This suggested that the uORF contained in the 5′UTR was suppressively working on the translation of the downstream ORF.

Deletion of the region containing SL1 resulted in increase of GUS activity by more than 2.2-fold due to lack of uORF and SL1 (Figure 1B, OsMac3(ΔSL1)). This result revealed that SL2 has some ability significantly enhancing the translation efficiency of the downstream ORF. We named the sequence of this region “dMac3” and suggest that dMac3 acts a strong translational enhancer. This efficiently supports translation initiation of the downstream ORF, so we attempted to analyze the dMac3 sequence and identify regions involved in its enhancer activity.

### Does dMac3 mRNA translation initiate via a 5′end-dependent mechanism?

**Figure 2:**
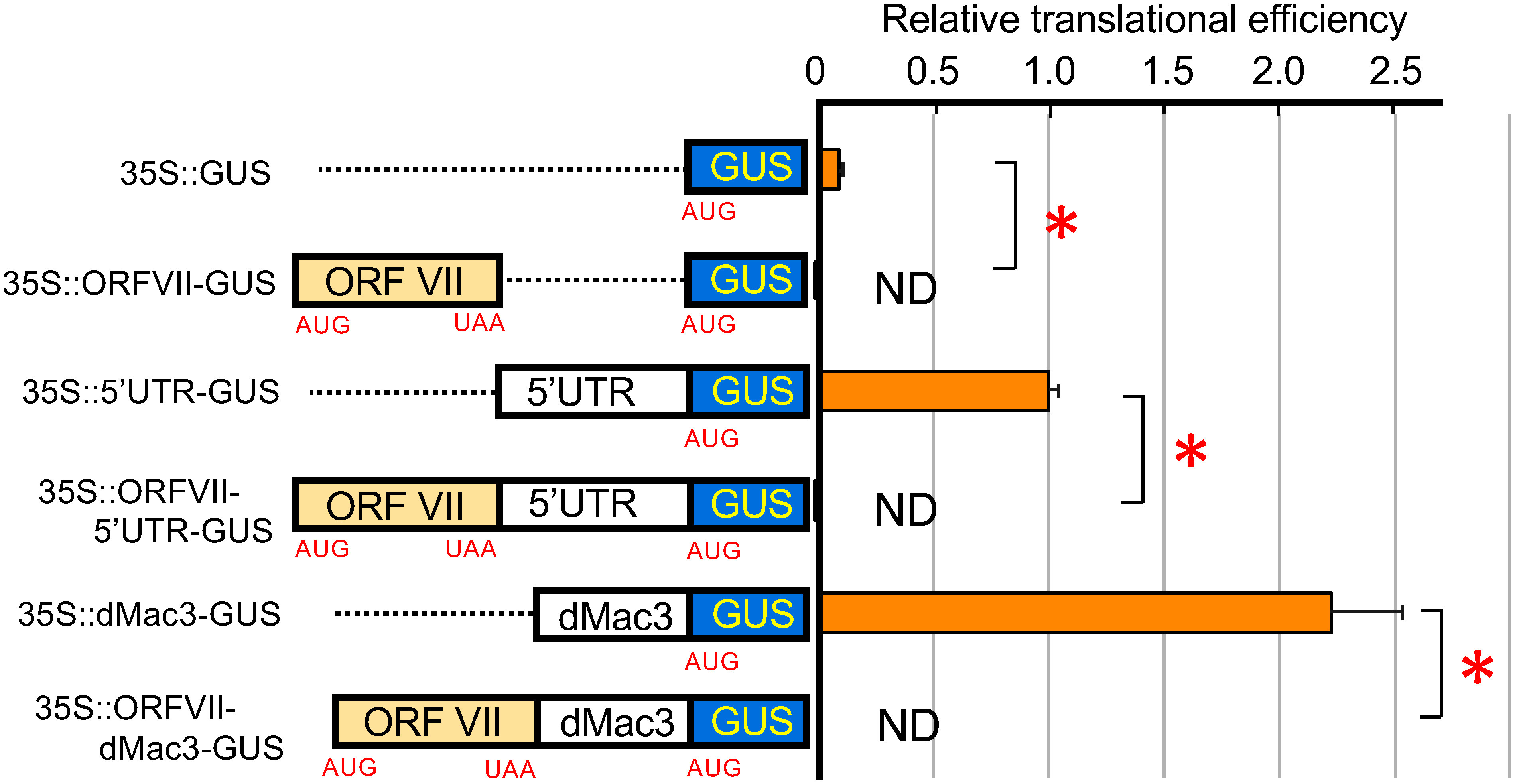
Figure 2. Examination of the IRES function in 5′UTR and dMac3 in the *OsMac3* mRNA. Left panels indicate the schematic representation of the reporter genes. *ORFVII* was introduced into the head portion and resulted in formation of a polycistronic mRNA. All of transcripts are driven by the CaMV *35S* promoter. Right panels show the relative translational efficiencies that were expressed as the ratio of the enzymatic activity of GUS. The relative translational efficiency was normalized against the *GUS* mRNA. The value of that with *35S::5′UTR-GUS* is set as 1.0. ND: not detected. Error bars indicate the SD (*n*=3). Asterisks indicate significant differences at *p*<0.01. The raw data of the GUS activity, relative GUS activity, and relative translational efficiency are shown in Supplementary Table S1.

First, we tested whether initiation on *dMac3-GUS* is dependent on the 5′-end structure or IRES. We analyzed the GUS activity in rice transformants and determined changes in translational efficiency by comparison with that of *35S::GUS*. It has been shown that translation initiation at *35S::GUS* depends on the 5′-end structure (Mutsuro-Aoki et al. 2021). We detected that translation initiation was blocked if a long ORFVII is placed upstream of the *GUS* ORF (Figure 2, 35S::ORFVII-GUS). Our transient expression experiments in rice protoplasts revealed that *GUS* ORF is efficiently recognized in the *OsMac3* *5′UTR-GUS* and *dMac3-GUS* (Figure 2, 35S::5′UTR-GUS, 35S::dMac3-GUS), but not in *ORFVII-5′UTR-GUS* and *ORFVII-dMac3-GUS* (Figure 2, 35S::ORFVII-5′UTR-GUS, 35S::ORFVII-dMac3-GUS). Thus, these suggest that dMac3 does not contain IRES that binds ribosomes directly, suggesting 5′-end dependent translation of dMac3 mRNAs.

### Dissection and mutational analysis of the dMac3 5′UTR

We assessed the translation efficiency of dMac3 or its mutant versions placed upstream of the *GUS* ORF. dMac3 contains a small SL (nts 14–34), SL2 (nts 44–127), and an unstructured region (nts 128–158) below SL2.

**Figure 3:**
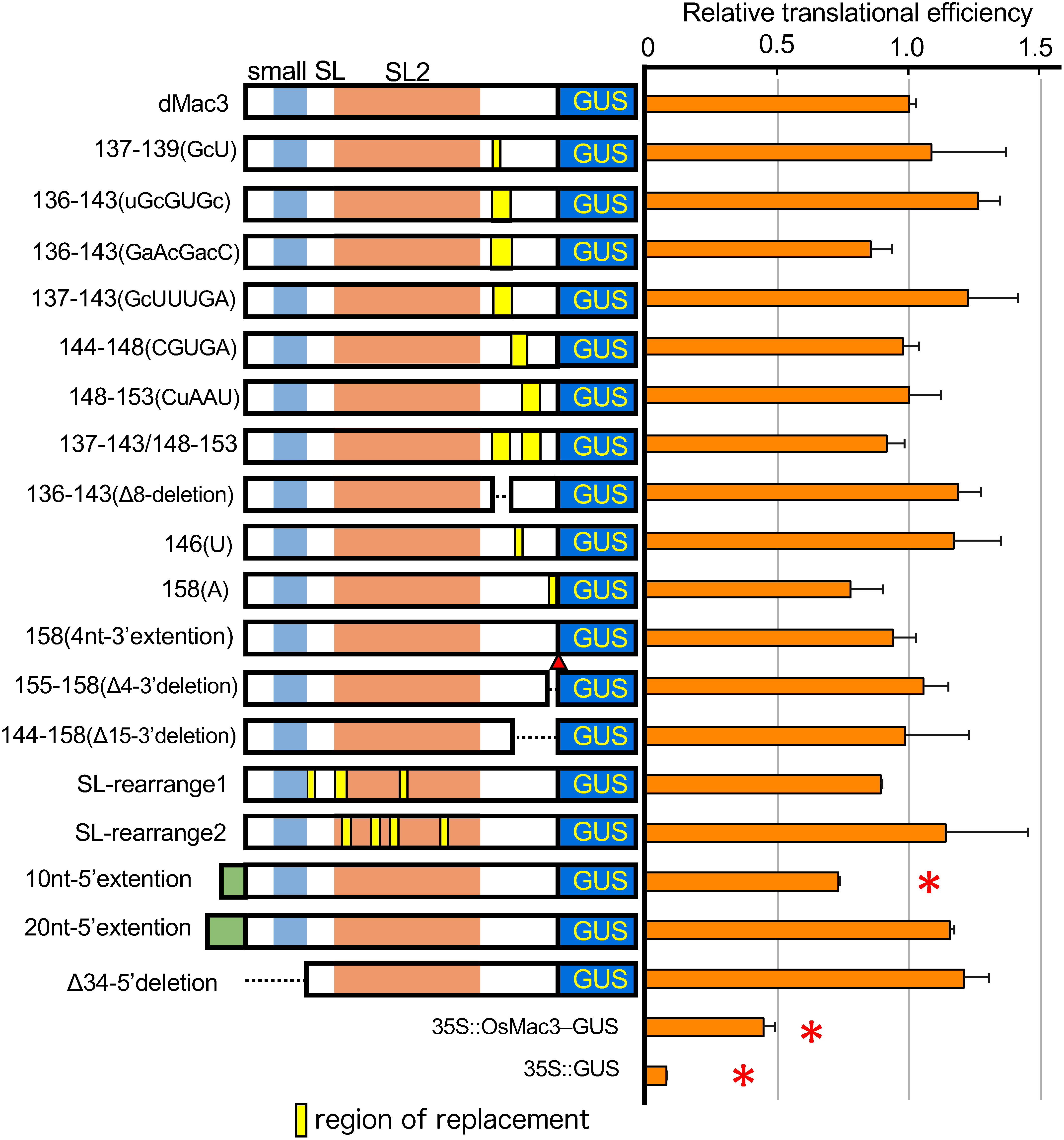
Figure 3. Translational efficiency of the *GUS* ORF with the modified dMac3. The left panel indicate the schematic representation of the structures of the mutant dMac3 whose nucleotide sequences are shown in Supplementary Figures S2 and S3. Numbers in the names of mutant genes indicate the regions of nucleotides that are substituted in dMac3. Changed nucleotides are indicated by capital letters in the nucleotide sequences shown in the parentheses. Regions for small SL, SL2 and the region of substitutions are indicated by pale blue, orange and yellow boxes. Deleted regions are indicated by broken lines. Added nucleotides are shown by red triangle or pale green boxes. *GUS* ORF is indicated by blue box with “GUS”. They were expressed using the *35S* promoter. Right panel indicates the relative GUS activities normalized against the *GUS* mRNA. The relative translational efficiency was normalized against the *GUS* mRNA. The value of the GUS activity with that of the reporter gene containing dMac3 is set as 1.0. The results represent the means of three independent experiments. Error bars indicate the SD (*n*=3). Asterisks indicate significant differences in the translational efficiency to that of dMac3 at *p*<0.01. Because the values varied depending on the viability of the cells used in each experiment, the relative GUS activities were normalized against the value of that of dMac3 in a simultaneous experiment. The raw data of the GUS activity, relative GUS activity, and relative translational efficiency are shown in Supplementary Table S1.

First, we prepared a series of mutations within nucleotides 137–158 (Figure 3) and analyzed their effect on transient expression in rice protoplasts. No significant difference in the translation efficiency was detected on the following mutants containing nucleotide substitutions in the various positions: 137–139(GcU), 136–143(uGcGUGc), 136–143(GaAcGacC), 137–143(GcUUUGA), 144–148(CGUGA), 148–153(CuAAu), 137–143/148–153, 136–143(Δ8-deletion), and 146(U) (Figure 3). Mutants, 158(A) where we replaced U with A at the 3′ end, 158(4nt-3′extension) where 4 bases at the dMac3 end were added, 155–158(Δ4-3′deletion) and 144–158(Δ15-3′deletion), lacking last 4 and 15 nucleotides in the 3′region of dMac3, respectively, showed similar translational efficiency to that of dMac3 (Figure 3). Overall, our substitution mutants as well as 3′ extensions or deletions did not significantly affect the level of GUS activity.

To analyze effect of mutations within the 5′ region of dMac3 on translation efficiency of *GUS* ORF, we prepared several extensions of 10 and 20 nucleotides at the 5′ proximity of dMac3 (10nt-5′extension and 20nt-5′extension; Figure 3). Evaluation of the translation efficiency of these mutants showed that 10nt-5′extension indicated smaller translational efficiency whereas that of 20nt-5′extension showed a similar value equivalent to that of dMac3 (Figure 3). The mutant Δ34-5′deletion, in which 34-nt in the 5′ region containing small SL was deleted, showed no significant difference in translation efficiency from that of dMac3 (Figure 3).

Next, we accomplished mutations within SL2, SL2-rearrange1 and SL2 rearrange2 (Supplementary Figure S3), that led to some rearrangement or change in stability of SL2. However, their translation efficiencies showed similar values to that of dMac3 (Figure 3).

### Evaluation of translation efficiency using vectors containing dMac3 driven by the *SPK* promoter or *RUBQ2* promoters

**Figure 4:**
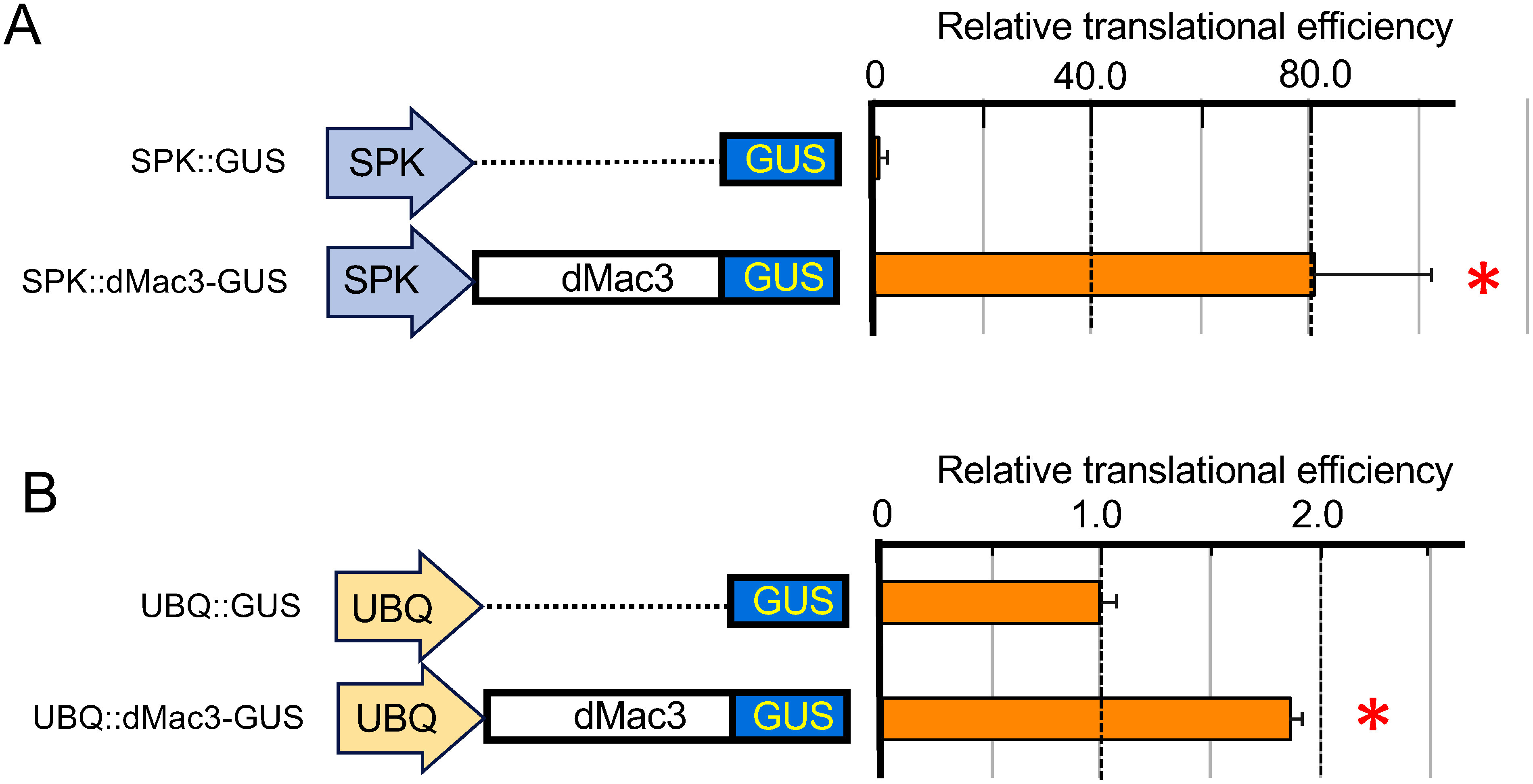
Figure 4. Translational efficiency of the mRNAs that are driven by the rice *SPK* promoter (SPK) and rice *RUBQ2* promoter (UBQ). Right panel indicates the relative translational efficiency normalized against the *GUS* mRNA, whose amount was estimated by real-time qRT-PCR. The value of the GUS activity with that of *SPK::GUS* or *UBQ::GUS* is set as 1.0. The results represent the means of three independent experiments. Error bars indicate the SD (*n*=3). Asterisks indicate significant differences in the translational efficiency to that of *SPK::GUS* or *UBQ::GUS* at *p*<0.01. The raw data of the GUS activity, relative GUS activity, and relative translational efficiency are shown in Supplementary Table S1. Amounts of transcripts of the reporter gene were shown in Supplementary Figure S4.

We investigated the translation efficiency of the transcripts where 5′UTR was placed under the *35S* promoter. Here we tested translation efficiency of the dMac3-GUS construct driven by either the *SPK* promoter or *RUBQ2* promoter, SPK::dMac3-GUS and UBQ::dMac3-GUS. These constructs were used to evaluate their translation efficiencies depending on the amount of mRNA and compared with those without dMac3. In these examinations, the yield of these transcripts was occasionally reduced due to unknown reasons. In those experiments where transcripts were driven by the *SPK* and *RUBQ2* promoters, the presence of dMac3 resulted in approximately 80- and 2-fold higher translation efficiency than in the absence of dMac3 (Figure 4A, B, respectively), confirming the high activity of the dMac3 5′UTR. These results indicate that the transcripts driven by these promoters show significantly high translational efficiency as similar to those driven by the *35S* promoter.

### Assessment of the influence of dMac3 on translation initiation in in vitro cell-free protein synthesis system and in the transient expression system

**Figure 5:**
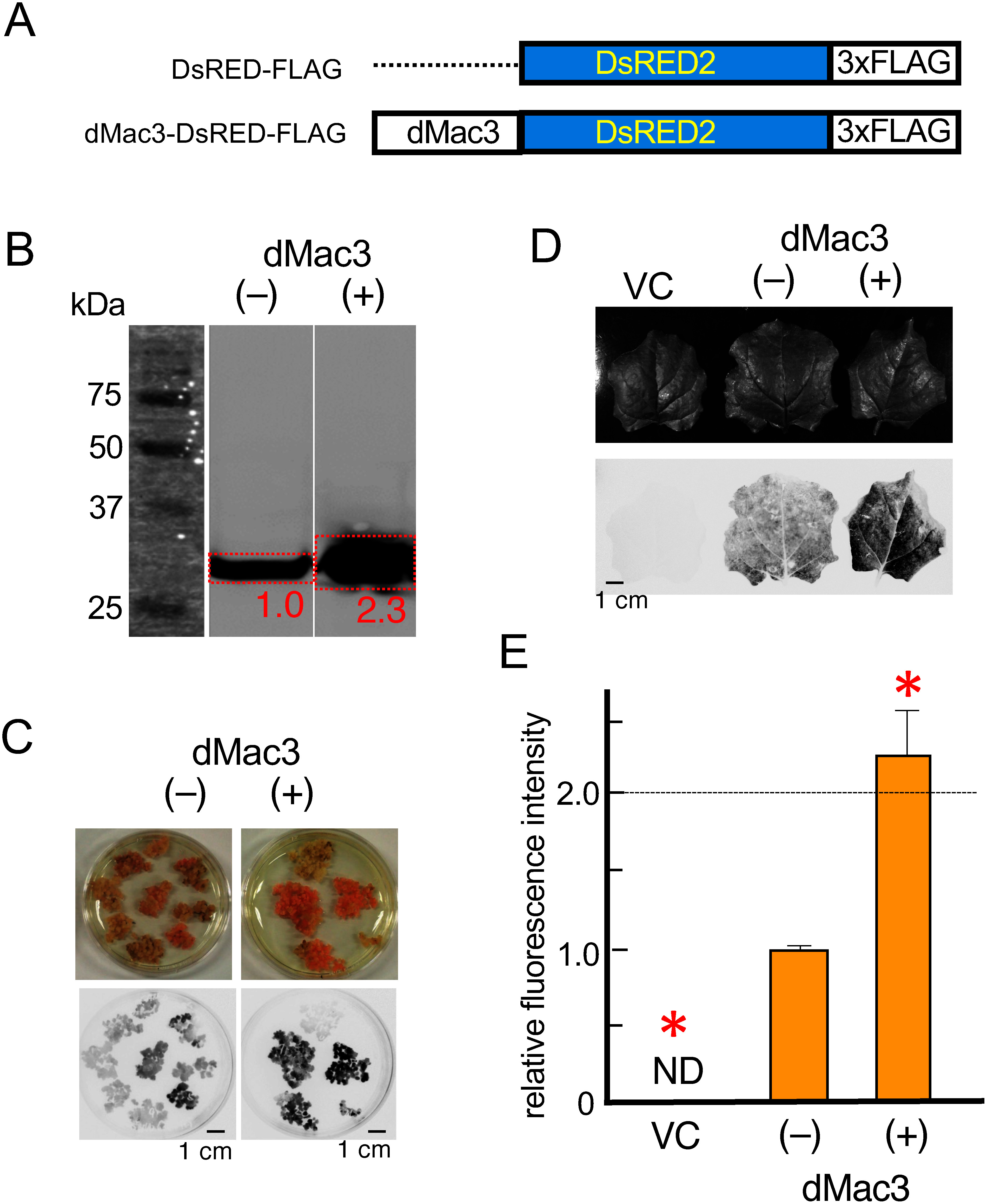
Figure 5. Protein production by in vitro translation system and by transient expression in tobacco leaves. (A) Schematic representation of the structures of the reporter *DsRED2* genes, which encoded a fusion protein of *DsRED2* with 3xFLAG tag. (B) in vitro production of the DsRED2 protein. DsRED2 fused with 3xFLAG tag was detected by the DsRED2 antiserum. (−) and (+) indicate the reactions containing *DsRED2-FLAG* and *dMac3-DsRED2-FLAG*, respectively. Size markers are shown in the left. The relative amounts of intensities of FLAG-tagged DsRED2 in the red-boxed regions are shown. (C) Detection of DsRED2 in rice cultured cells. Callus were transformed by *DsRED2-FLAG* (−) and *dMac3-DsRED2-FLAG* (+). The upper panels show the visible features of representative tranformant calli and the lower panels show their fluorescence images. (D) Transient expression of FLAG-tagged DsRED2 in tobacco leaves. VC: pCAMBIA1301 (vector control), CT: *DsRED2-FLAG* (−), dMac3: *dMac3-DsRED2-FLAG* (+). Upper and lower panels show the visible features of representative transformant leaves and their fluorescence images, respectively. (E) Relative fluorescence intensities of the transformant leaves that were created independently. The value of CT was set as 1.0. Error bars indicate the SD (*n*=3). Asterisks indicate significant differences to that of CT at *p*<0.01.

We also used a cell-free protein synthesis system derived from wheat germs (Sawasaki et al. 2002) to directly analyze the effect of *dMac3 5′UTR* on translation of *dMac3*-containing mRNAs. Thus, we constructed reporter constructs containing or not *dMac3 5′UTR* (*DsRED2-FLAG* and *dMac3-DsRED2-FLAG*) both encoding the FLAG-tagged protein DsRED2, for translation in wheat germ in vitro system (Figure 5A). *DsRED2-FLAG* mRNAs with or without *dMac3 5′UTR* were synthesized in vitro transcription system as described in Materials and methods. mRNAs containing or not dMac3-5′UTR were translated in parallel in wheat germ extract, followed by analysis of the synthesized product by Western blotting using anti-FLAG antibodies. This analysis revealed that dMac3-DsRED2-FLAG resulted in 2.3-fold amount of DsRED2 production (Figure 5B). This shows that dMac3 has a positive effect on 3xFLAG-tagged DsRED2 protein (29 kDa) production in wheat germ, increasing its synthesis significantly due to the introduction of dMac3 into the 5′UTR of mRNA. This result indicates that dMac3 satisfactorily functions in the wheat germ in vitro protein production system, and that protein production is efficiently increased by introducing dMac3 into the 5′UTR of mRNA.

We next attempted to produce 3xFLAG-tagged DsRED2 in cultured rice cells using either the *35S::DsRED2-FLAG* or *35S::dMac3-DsRED2-3FLAG* containing vector. *35S::dMac3-DsRED2-3FLAG* produced larger amount of protein than that produced by *DsRED2-FLAG* that did not contain dMac3 (Figure 5C). These genes were introduced into the green leaves of *Nicotiana benthamiana* by the transient expression using *Agrobacterium* infiltration method. Again, levels of DsRED2 by *DsRED2-FLAG* were higher than when the vector construct did not contain dMac3 (Figure 5D). We found that production of FLAG-tagged DsRED2 was 2.2-fold higher for the *35S::dMac3-DsRED2-FLAG* vector compared to *35S::DsRED2-FLAG* (Figure 5E).

## Discussion

The 5′UTR of *OsMac3* showed enhanced translational efficiency in the downstream ORF. This suggests that this region has some mechanism leading increased translation of the downstream ORF. The 5′UTR of *OsMac3* contained an uORF and was predicted to have two regions for stem-and-loop structures SL1 and SL2 (Figure 1A). A mutant 5′UTR lacking this uORF showed significant increase in translation of the downstream ORF (Figure 1B). This suggests that the uORF has a negative effect on the downstream ORF translation.

The uORF starts from AUG within SL1. We created two mutants lacking the SL2 region, one of which contained AUG derived from the uORF and in the other of which this AUG was replaced by AAG. The mutant with AUG, OsMac3(ΔSL2), had an ORF continuing to the region for the downstream ORF using a different frame from that of the GUS gene. OsMac3(ΔSL2) mutant containing AUG showed no GUS activity, whereas the other mutant lacking this AUG, OsMac3(ΔSL2ΔAUG), showed sufficient GUS activity indicating a high translation efficiency (Figure 1B). This result suggests that translation occurred from the AUG in the SL2 mutant, and this event inhibited the translation of the downstream ORF. Thus, the knockout of AUG can further increase the high translational enhancing activity of *OsMac3* 5′UTR.

A mutant lacking SL1, named dMac3, showed significantly higher translational efficiency in the downstream ORF. dMac3 consists of 158 nt region of the 3′ region of the *OsMac3* 5′UTR. In this case, the level of translation was 2–3 fold higher than that of the entire 5′UTR (Figure 1). This fact suggests that dMac3 presumably achieves more than 9 times higher promotion of the translational efficiency in the downstream ORF as compared to that without *OsMac3* 5′UTR (Figure 1B). This indicates that dMac3 is very useful as an excellent translation enhancer for the downstream ORF.

Some viral mRNAs are known to have IRES sequences preceding the downstream ORFs. We determined whether the IRES sequence exists in 5′UTR. When another ORF, such as ORFVII, was inserted upstream *OsMac3* 5′UTR or dMac3, translation of the downstream ORF was no longer observed (Figure 2). These results strongly suggested that mRNAs containing these sequences were translated in a 5′-end dependent manner. This indicates that *OsMac3* 5′UTR and dMac3 does not have a sequence with IRES activity.

It has been shown that some eukaryotic mRNAs exhibit a ribosome shunting mechanism that promotes the translation of the downstream ORF. In this case, a specific region downstream the stem-loop structure may play an important role in determining translation efficiency (Yueh and Schneider 2000). dMac3 was predicted to have a small SL in the 14–34 nt region and an SL2 in the 44–127 nt region. Translation from the mRNA containing *OsMac3* 5′UTR as well as dMac3 was suggested to occur in the 5′ CAP dependent manner, and therefore we assumed that there might be an mRNA shunting mechanism in such a long 5′UTR to achieve an efficient translation of the downstream ORF.

If ribosome shunting occurs within dMac3, the region immediately downstream of SL2 could be important. By modifying this region, we created dMac3 mutants and analyzed their effect on the translation of the downstream ORF. However, mutants containing nucleotide substitutions in the 136–158 nt region corresponding to the 3′ region of dMac3 showed no significant changes in translation efficiency, indicating that none of these mutants impair the translation efficiency of the downstream ORF (Figure 3). The mutants Δ15-3′deletion and Δ4-3′deletion that lacked the 15 nt in the 3′ end region showed the similar translation efficiency to that of dMac3 (Figure 3). Our results did not confirm that dMac3 involves a ribosome shunting mechanism during translation of *OsMac3* mRNA. Further analysis is necessary to understand the importance of this area.

In a mutant lacking SL2, the translation efficiency from mRNA was significantly reduced (Figure 1B), suggesting that this region is important for the translational efficiency in dMac3. However, no significant change was detected in the translational efficiency of mutants SL-arranges 1 and 2, in which the nucleotide sequence of the SL2 region was modified, although it was suggested that this region affects the stability of the secondary structure of dMac3 (Figure 3). These results imply that the nucleotide sequence in this region was not so critical for translational enhancer activity of dMac3. The reason why these differences occur is unclear.

**Figure 6:**
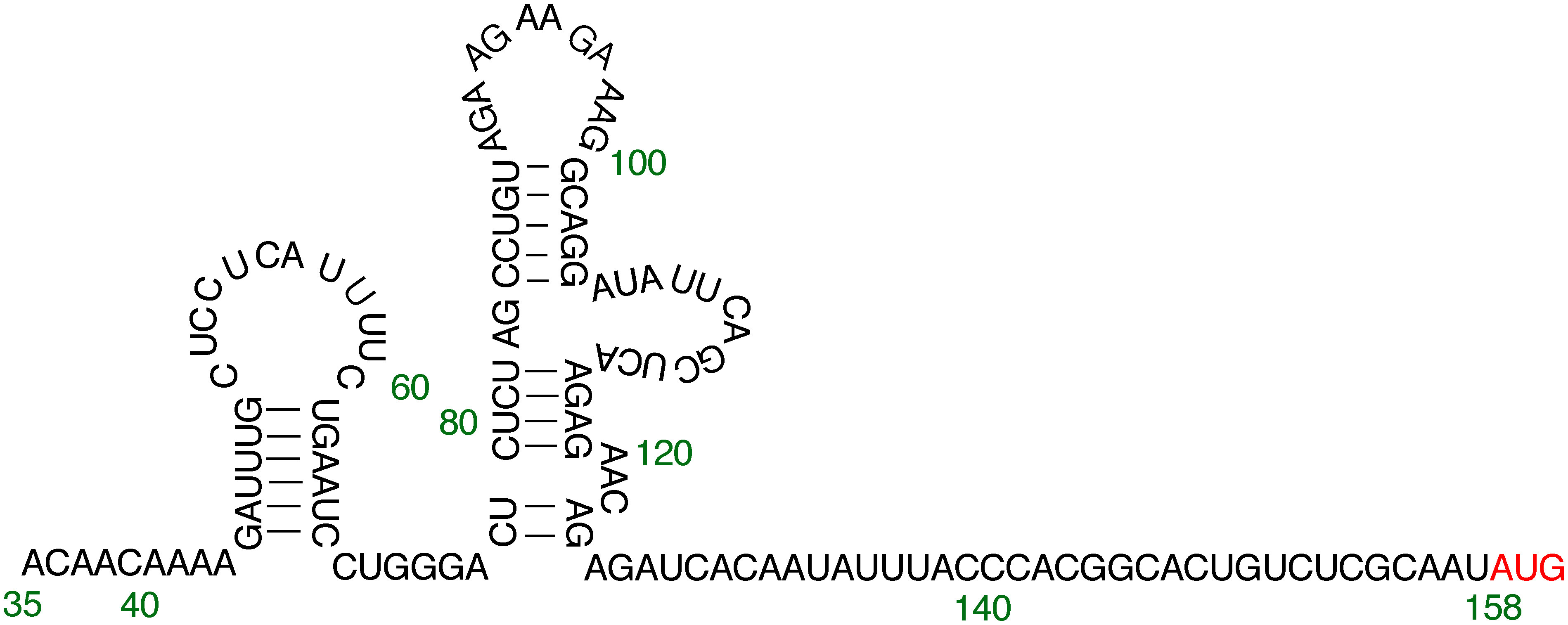
Figure 6. A predicted stem-loop structure that could form in the modified dMac3 region contained in Δ34-5′deletion mutant. Nucleotide numbers in dMac3 are indicated. The initiation codon of the downstream ORF is indicated by red letters.

The dMac3 mutant whose 5′ ends was extended by 10 nucleotides showed a reduced translational efficiency (Figure 3). On the other hand, the dMac3 mutant 20nt-5′extention and Δ34-5′deletion, which harbored 20 nt addition and the 34 nt deletion in the 5′ end of dMac3, showed similar translation efficiency than that of dMac3 (Figure 3). The Δ34-5′deletion mutant lost the region corresponding to small SL, suggesting less importance of this region in translation initiation. However, in this mutant, formation of another stem-loop structure was predicted in the 44–67 nt region (Figure 6). Although it is unclear whether the formation of such a secondary structure actually occurred in this mutant of dMac3, it is possible that some structural alteration may have led to reconstitute an efficient translation. We presume that the translational enhancement by dMac3 is not dependent on specific nucleotide sequences but rather on the overall conformation formed within the 5′UTR.

The effect of translational enhancer was also achieved for the mRNAs obtained using a promoter other than the 35S, such as *SPK* and *RUBQ2* promoters (Figure 4). Higher enhancer activity values were obtained using *SPK* rather than *RUBQ2*. SPK gene is highly expressed in immature seeds (Kawasaki et al. 1999), but its expression is very low in other cells (https://rapdb.dna.affrc.go.jp/locus/?name=Os10g0539600), suggesting that a small amount of mRNA will generate in the protoplast cells. Actually qRT-PCR detected a small amount of mRNA in the reaction solution (Supplementary Figure S4). On the other hand, ubiquitin gene is known to be highly expressed in various cells. Our results showed a significant difference in translation efficiency among the mRNA driven by SPK and ubiquitin promoters. From these results, we presume that the translation enhancer activity of dMac3 seems different depending on the mRNA species transcribed by different promoters. There might be some reason that causes differences in activity depending on the type of promoter used. We imagine that dMac3 may more efficiently work for a mRNA driven by a weak promoter but less efficiently for a mRNA driven by a strong promoter.

Cell-free protein synthesis systems using a wheat-germ extracts can easily synthesize a variety of proteins. We found that protein production by this synthesis was significantly improved when using dMac3 (Figure 5). When transiently expressed in cultured rice cells or *N. benthamiana* leaves, the downstream ORF was efficiently translated, and large amounts of protein were produced (Figure 5).

To date, several attempts have been made to construct genome editing vectors using dMac3, such as dMac3-TALEN system and CRISPR-dMac3-Cas9 system (Kusano et al. 2018; Onodera et al. 2018). Genome editing using these systems allows efficient targeted mutagenesis and creation of desired mutants (Ohnuma et al. 2023; Onodera et al. 2018; Takeuchi et al. 2021). These results are considered to be attributed to the effect of increasing the amount of TALEN and Cas9 due to dMac3. These results demonstrate that dMac3 is a high-performance translation enhancer that can be generally used in many different situations, where high protein levels are critical.

## Abbreviations

PCR: polymerase chain reaction
RT-PCR: reverse-transcriptase-mediated PCR

## Description of Supplementary Files

Supplementary Figure S1. Nucleotide sequences of the 5′UTR of *OsMac3* and its derivatives.

Supplementary Figure S2. Nucleotide sequences of dMac3 and its derivatives.

Supplementary Figure S3. Nucleotide sequence of SL-rearrange1 and SL-rearrange2.

Supplementary Figure S4. Amounts of transcripts of the reporter gene in the reaction solution.

Supplementary Table S1. The raw data of the GUS activity, relative GUS activity, and relative translational efficiency.
