# Supplementary material for: Identification of a minimal strong translation enhancer within the 5′-untranslated region of OsMac3 mRNA
Source: Plant Biotechnol (Tokyo). 2024 Dec 25;41(4):437–46. doi: 10.5511/plantbiotechnology.24.0909a (PMC11897733; doi:10.5511/plantbiotechnology.24.0909a)
Supplement: Supplementary Data [file plantbiotechnology-41-4-24.0909a-s001.pdf]

OsMac3

```
GAAGCGCCAGACAAATTCGT CTCCCTCGTTGGAAGTGGAG GCGCGGAGGCGGAAGCTTTT 60
CCTCCTCCTCTCCTCTCCTC TCTACCCTAGGCGCGATCTG CTCGTCCGCCTCGGCGATCC 120
ACAGGGAAGGAGCAGCATGC TCCACAAAGACGCACTACAG AAGACTAAAGAGAGCTTTTT 180
CATACCAAAGAAGTACAACA AAAGATTTGCTCCTCATTTT CTGAATCCTGGGACTCTCTA 240
GCCTGTAGAAGAAGAAAGGC AGGAATTTCAGCTCAAGAGA ACAGATCACAATATTTACCC 300
ACGGCACTGTCTCGCAAT
```

OsMac3(ΔuORF)

```
GAAGCGCCAGACAAATTCGT CTCCCTCGTTGGAAGTGGAG GCGCGGAGGCGGAAGCTTTT 60
CCTCCTCCTCTCCTCTCCTC TCTACCCTAGGCGCGATCTG CTCGTCCGCCTCGGCGATCC 120
ACAGGGAAGGAGCAGCAAGC TCCACAAAGACGCACTACAG AAGACTAAAGAGAGCTTTTT 180
CATACCAAAGAAGTACAACA AAAGATTTGCTCCTCATTTT CTGAATCCTGGGACTCTCTA 240
GCCTGTAGAAGAAGAAAGGC AGGAATTTCAGCTCAAGAGA ACAGATCACAATATTTACCC 300
ACGGCACTGTCTCGCAAT
```

OsMac3(ΔSL1) (dMac3)

```
-------------------- -------------------- -------------------- 60
-------------------- -------------------- -------------------- 120
-------------------- -------------------- AAGACTAAAGAGAGCTTTTT 180
CATACCAAAGAAGTACAACA AAAGATTTGCTCCTCATTTT CTGAATCCTGGGACTCTCTA 240
GCCTGTAGAAGAAGAAAGGC AGGAATTTCAGCTCAAGAGA ACAGATCACAATATTTACCC 300
ACGGCACTGTCTCGCAAT
```

OsMac3(ΔSL2)

```
GAAGCGCCAGACAAATTCGT CTCCCTCGTTGGAAGTGGAG GCGCGGAGGCGGAAGCTTTT 60
CCTCCTCCTCTCCTCTCCTC TCTACCCTAGGCGCGATCTG CTCGTCCGCCTCGGCGATCC 120
ACAGGGAAGGAGCAGCATGC TCCACAAAGACGCACTACAG AAGACTAAAGAGAGCTTTTT 180
CATACCAAAGAAGTACAACA AAA----------------- -------------------- 203
-------------------- ----ATTTCAGCTCAAGAGA ACAGATCACAATATTTACCC 239
ACGGCACTGTCTCGCAAT
```

OsMac3(ΔSL2ΔAUG)

```
GAAGCGCCAGACAAATTCGT CTCCCTCGTTGGAAGTGGAG GCGCGGAGGCGGAAGCTTTT 60
CCTCCTCCTCTCCTCTCCTC TCTACCCTAGGCGCGATCTG CTCGTCCGCCTCGGCGATCC 120
ACAGGGAAGGAGCAGCAAGC TCCACAAAGACGCACTACAG AAGACTAAAGAGAGCTTTTT 180
```

```
CATACCAAAGAAGTACAACA AAA----------------- -------------------- 203
-------------------- ----ATTTCAGCTCAAGAGA ACAGATCACAATATTTACCC 239
ACGGCACTGTCTCGCAAT
```

Supplementary Figure S1. Nucleotide sequences of the 5'UTR of *OsMac3* and its derivatives. The region for the initiation codon of uORF and the modified one are shown by red letters. Gaps indicate the deleted regions.

dMac3

AAGACTAAAGAGAGCTTTTT CATACCAAAGAAGTACAACA AAAGATTTGCTCCTCATTTT
CTGAATCCTGGGACTCTCTA GCCTGTAGAAGAAGAAAGGC AGGAATTTCAGCTCAAGAGA
ACAGATCACAATATTTACCC ACGGCACTGTCTCGCAAT

137-139(GcU)

AAGACTAAAGAGAGCTTTTT CATACCAAAGAAGTACAACA AAAGATTTGCTCCTCATTTT
CTGAATCCTGGGACTCTCTA GCCTGTAGAAGAAGAAAGGC AGGAATTTCAGCTCAAGAGA
ACAGATCACAATATTTGCTC ACGGCACTGTCTCGCAAT

136-143(uGcGUGc)

AAGACTAAAGAGAGCTTTTT CATACCAAAGAAGTACAACA AAAGATTTGCTCCTCATTTT
CTGAATCCTGGGACTCTCTA GCCTGTAGAGAAGAAAGGCA GGAATTTCAGCTCAAGAGAA
CAGAUCACAATATTTTGCGT GCGGCACTGTCTCGCAAT

136-143(GaAcGacC)

AAGACTAAAGAGAGCTTTTT CATACCAAAGAAGTACAACA AAAGATTTGCTCCTCATTTT
CTGAATCCTGGGACTCTCTA GCCTGTAGAAGAAGAAAGGC AGGAATTTCAGCTCAAGAGA
ACAGATCACAATATTGAACG ACCGCACTGTCTCGCAAT

137-143(GcUUUGA)

AAGACTAAAGAGAGCTTTTT CATACCAAAGAAGTACAACA AAAGATTTGCTCCTCATTTT
CTGAATCCTGGGACTCTCTA GCCTGTAGAAGAAGAAAGGC AGGAATTTCAGCTCAAGAGA
ACAGATCACAATATTTGCTT TGAGCACTGTCTCGCAAT

144-148(CGUGA)

AAGACTAAAGAGAGCTTTTT CATACCAAAGAAGTACAACA AAAGATTTGCTCCTCATTTT
CTGAATCCTGGGACTCTCTA GCCTGTAGAAGAAGAAAGGC AGGAATTTCAGCTCAAGAGA
ACAGATCACAATATTTACCC ACGCGTGAGTCTCGCAAT

148-153(CuAAU)

AAGACTAAAGAGAGCTTTTT CATACCAAAGAAGTACAACA AAAGATTTGCTCCTCATTTT
CTGAATCCTGGGACTCTCTA GCCTGTAGAAGAAGAAAGGC AGGAATTTCAGCTCAAGAGA
ACAGATCACAATATTTACCC ACGGCACTCTAATGCAAT

137-143/148-153

AAGACTAAAGAGAGCTTTTT CATACCAAAGAAGTACAACA AAAGATTTGCTCCTCATTTT
CTGAATCCTGGGACTCTCTA GCCTGTAGAAGAAGAAAGGC AGGAATTTCAGCTCAAGAGA
ACAGATCACAATATTTGCTT TGAGCACTCTAATTCAAT

136-143(Δ8-deletion)
AAGACTAAAGAGAGCTTTTT CATACCAAAGAAGTACAACA AAAGATTTGCTCCTCATTTT
CTGAATCCTGGGACTCTCTA GCCTGTAGAAGAAGAAAGGC AGGAATTTCAGCTCAAGAGA
ACAGATCACAATATT----- ---GCACTGTCTCGCAAT

146(U)
AAGACTAAAGAGAGCTTTTT CATACCAAAGAAGTACAACA AAAGATTTGCTCCTCATTTT
CTGAATCCTGGGACTCTCTA GCCTGTAGAAGAAGAAAGGC AGGAATTTCAGCTCAAGAGA
ACAGATCACAATATTTACCC ACGGCTCTGTCTCGCAAT

158(A)
AAGACTAAAGAGAGCTTTTT CATACCAAAGAAGTACAACA AAAGATTTGCTCCTCATTTT
CTGAATCCTGGGACTCTCTA GCCTGTAGAAGAAGAAAGGC AGGAATTTCAGCTCAAGAGA
ACAGATCACAATATTTACCC ACGGCACTGTCTCGCAAA

4nt-3'extention
AAGACTAAAGAGAGCTTTTT CATACCAAAGAAGTACAACA AAAGATTTGCTCCTCATTTT
CTGAATCCTGGGACTCTCTA GCCTGTAGAAGAAGAAAGGC AGGAATTTCAGCTCAAGAGA
ACAGATCACAATATTTACCC ACGGCACTGTCTCGCAATCGTA

155-158(Δ4-3'deletion)
AAGACTAAAGAGAGCTTTTT CATACCAAAGAAGTACAACA AAAGATTTGCTCCTCATTTT
CTGAATCCTGGGACTCTCTA GCCTGTAGAAGAAGAAAGGC AGGAATTTCAGCTCAAGAGA
ACAGATCACAATATTTACCC ACGGCACTGTCTCG....

144-158(Δ15-3'deletion)
AAGACTAAAGAGAGCTTTTT CATACCAAAGAAGTACAACA AAAGATTTGCTCCTCATTTT
CTGAATCCTGGGACTCTCTA GCCTGTAGAAGAAGAAAGGC AGGAATTTCAGCTCAAGAGA
ACAGATCACAATATTTACCC ACG...............

SL-reaarange1
AAGACTAAAGAGAGCTTTTT CATACCAAAAAAGCACAACA AAAAGCAGACTCCTCATTTT

CTGAATCCTGGGACTACATA GCCTGTAGAAGAAGAAAGGC AGGAATTTCAGCTCAAGAGA
ACAGATCACAATATTTACCC ACGGCACTGTCTCGCAAT

SL-reaarange2
AAGACTAAAGAGAGCTTTTT CATACCAAAGAAGTACAACA AAAGATTTCATCCTCATTTT
CTGAATAACGGGTAACTCTA GCCTGTAGAAGAAGAAAGGC AGAAATTTCAGCTCAAGAGA
ACAGATCACAATATTTACCC ACGGCACTGTCTCGCAAT

10nt-5'extention
CGCACTACAGAAGACTAAAG AGAGCTTTTTCATACCAAAG AAGTACAACAAAAGATTTGC
TCCTCATTTTCTGAATCCTG GGACTCTCTAGCCTGTAGAA GAAGAAAGGCAGGAATTTCA
GCTCAAGAGAACAGATCACA ATATTTACCCACGGCACTGT CTCGCAAT

20nt-5'extention
TCCACAAAGACGCACTACAG AAGACTAAAGAGAGCTTTTT CATACCAAAGAAGTACAACA
AAAGATTTGCTCCTCATTTT CTGAATCCTGGGACTCTCTA GCCTGTAGAAGAAGAAAGGC
AGGAATTTCAGCTCAAGAGA ACAGATCACAATATTTACCC ACGGCACTGTCTCGCAAT

Δ34-5'deletion
-------------------- --------------ACAACA AAAGATTTGCTCCTCATTTT
CTGAATCCTGGGACTCTCTA GCCTGTAGAAGAAGAAAGGC AGGAATTTCAGCTCAAGAGA
ACAGATCACAATATTTACCC ACGGCACTGTCTCGCAAT

Supplementary Figure S2. Nucleotide sequences of dMac3 and its derivatives. Red and blue letters indicate the nucleotides that are substituted from those of dMac3 and added to dMac3, respectively. Gaps indicate the deleted regions. Inserted nucleotides are shown by blue letters.

SL-rearrange1

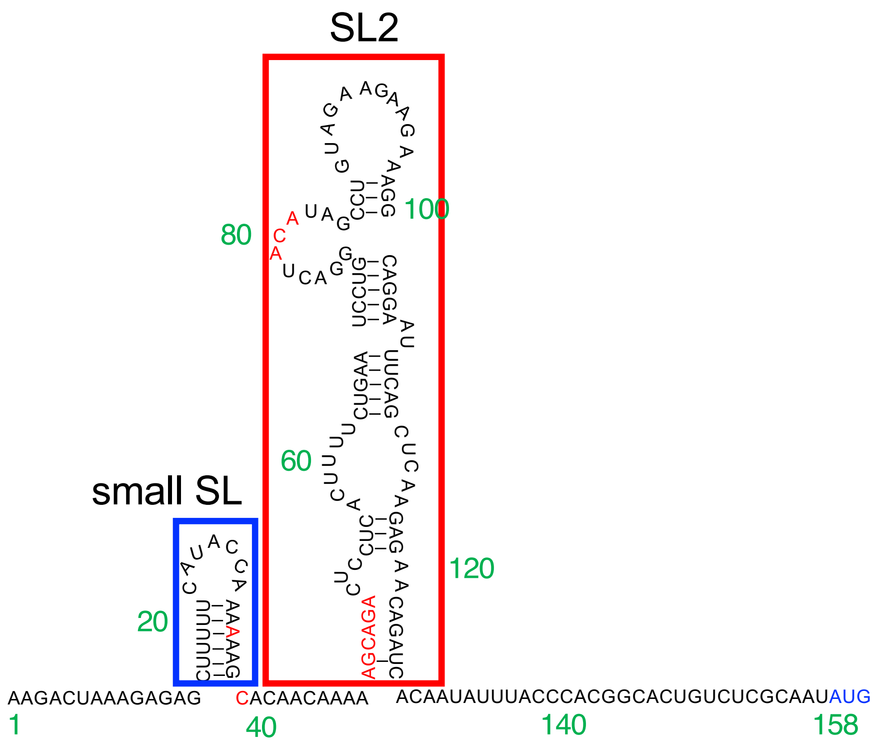

SL-rearrange2

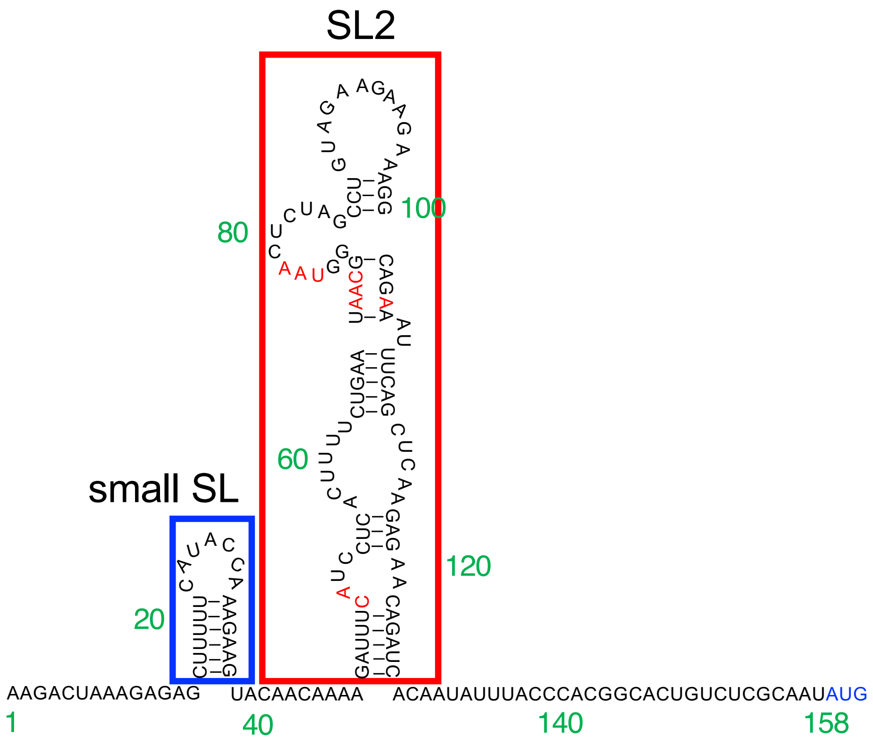

Supplementary Figure S3. Nucleotide sequence of SL-rearrange1 and SL-rearrange2. The substituted nucleotides are shown by red letters on the figure of the predicted secondary structure of dMac3.

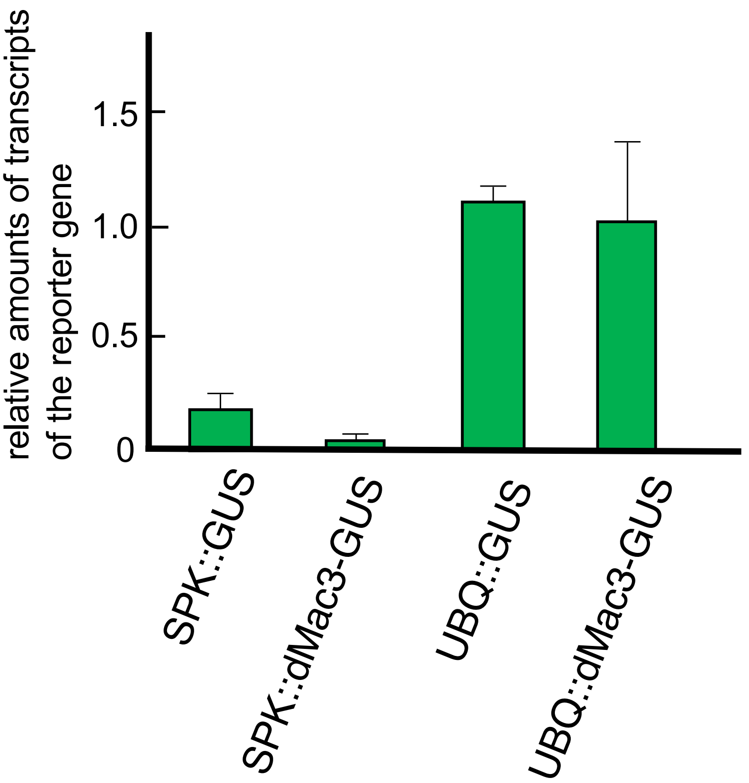

Supplementary Figure S4. Amounts of transcripts of the reporter gene in the reaction solution (average of three reaction samples). Relative amount of mRNA was measured by realtime RT-PCR using Thunderbird SYBR qPCR mix (Toyobo, Osaka, Japan). Error bars indicate the SD of three reaction samples. The value of UBQ::dMac3-GUS was set as 1.0.

Figure 1B	Sample		GUS activity (Raw)	Value of Cell factor	Relative GUS activity	SD	Translational efficiency	SD
	35s::GUS	1	288544	6515.3	44.3		0.198	
		2	264682	7775.3	34.0		0.152	
		3	238025	6589.3	36.1		0.162	
		average	263750	6960.0	38.2	5.42	0.171	0.0198
	OsMac3	1	2417803	11105.3	217.7		1.030	
		2	1818813	10685.3	170.2		0.856	
		3	2065712	7319.3	282.2		1.114	
		average	2100776	9703.3	223.4	56.2	1.000	0.2054
	OsMac3(ΔuORF)	1	4726634	10535.3	448.6		2.008	
		2	4582838	12858.3	356.4		1.595	
		3	5533830	13292.3	416.3		1.864	
		average	4947767	12228.7	407.1	46.8	1.823	0.1711
	OsMac3(ΔSL1)	1	614205	1246.6	492.7		2.206	
	(dMac3)	2	1304233	2436.0	535.4		2.397	
		3	1050109	2158.1	486.6		2.178	
		average	989516	1946.9	504.9	19.6	2.260	1.9723
	OsMac3(ΔSL2)	1	3820	7689.3	0.5		0.002	
		2	5834	10199.3	0.6		0.002	
		3	14105	9133.3	1.5		0.006	
		average	7920	9007.3	0.9	0.58	0.004	0.0019
	OsMac3(ΔSL2ΔAUG)	1	103867	396.7	261.84		1.225	
		2	117309	794.7	147.62		0.691	
		3	111245	678.7	163.92		0.767	
		average	110807	623.3	191.13	61.8	0.894	0.2361

Figure 2	Sample		GUS activity (Raw)	Value of Cell factor	Relative GUS activity	SD	Translational efficiency	SD
	35S::GUS	1	14192	862.7	16.5		0.077	
		2	12757	828.7	15.4		0.072	
		3	10964	452.7	24.2		0.113	
		average	12638	714.7	18.7	4.8199	0.087	0.0226
	35S::ORFVII-GUS	1	-396	1142.7	-0.3		-0.002	
		2	-1646	698.7	-2.4		-0.011	
		3	-2619	670.7	-3.9		-0.018	
		average	-1554	837.3	-2.2	1.78	-0.010	0.0084
	35S::5'UTR-GUS	1	106121	478.7	221.7		1.038	
		2	105448	522.7	201.8		0.944	
		3	74119	340.7	217.6		1.018	
		average	95229	447.3	213.7	10.5	1.000	0.0493
	35S::ORFVII-5'UTR-GUS	1	-462	410.7	-1.1		-0.005	
		2	813	820.7	1.0		0.005	
		3	-686	572.7	-1.2		-0.006	
		average	-112	601.3	-0.4	1.24	-0.002	0.0058
	35S::dMac3-GUS	1	152850	376.7	405.8		1.899	
		2	199397	352.7	565.4		2.646	
		3	225695	496.7	454.4		2.127	
		average	192647.3333	408.7	475.2	81.81	2.224	0.3829
	35S::ORFVII-dMac3-GUS	1	-834	874.7	-0.9535		-0.004	
		2	193	1026.7	0.1880		0.001	
		3	1175	1348.7	0.8712		0.004	
		average	178	1083.3	0.0352	0.92	0.000	0.0043

Figure 3	Sample		GUS activity (Raw)	Value of Cell factor	Relative GUS activity	SD	Translational efficiency	SD
	dMac3	1	614205	1696.0	362.1		0.9758	
		2	1304233	3314.0	393.6		1.0604	
		3	1050109	2936.0	357.7		0.9637	
		average	989516	2648.7	371.1	19.6	1.0000	0.0527

137-139(GcU)	1	807699	3062.6	263.7		0.7106	
	2	1354214	3190.3	424.5		1.1438	
	3	2115504	4084.3	518.0		1.3956	
	average	1425805	3445.7	402.1	264.89	1.0833	0.3465
136-143(uGcGUGc)	1	611586	1264.0	483.8		1.3037	
	2	761032	1798.0	423.3		1.1405	
	3	583067	1180.0	494.1		1.3314	
	average	651895	1414.0	467.1	38.3	1.2586	0.1032
136-143(GaAcGacC)	1	740293	2094.0	353.5		0.9526	
	2	621013	1962.0	316.5		0.8529	
	3	541152	1934.0	279.8		0.7540	
	average	634153	1996.7	316.6	36.9	0.8531	0.0993
137-143(GcUUUGA)	1	532616	1449.0	367.6		0.9905	
	2	539271	1174.1	459.3		1.2376	
	3	585806	1090.9	537.0		1.4469	
	average	552564	1238.0	454.6	84.8	1.2250	0.2285
144-148(CGUGA)	1	5134081	13096.1	392.0		1.0563	
	2	8067534	23773.8	339.3		0.9144	
	3	7769062	21737.2	357.4		0.9630	
	average	6990226	19535.7	362.9	16.3	0.9779	0.0721
148-153(CuAAU)	1	868727	2126.0	408.6		1.1010	
	2	827766	2088.0	396.4		1.0682	
	3	559303	1816.0	308.0		0.8299	
	average	751932	2010.0	371.0	54.9	0.9997	0.1480
137-143/148-153	1	316007	862.0	366.6		0.9878	
	2	409970	1186.0	345.7		0.9314	
	3	330257	1068.0	309.2		0.8332	
	average	352078	1038.7	340.5	29.0	0.9175	0.0782
136-143(Δ8-deletion)	1	985139	2079.1	473.8		1.2768	
	2	659001	1658.2	397.4		1.0708	
	3	755445	1682.1	449.1		1.2102	
	average	799861	1806.5	440.1	77.4	1.1859	0.1051
146(U)	1	827582	1700.0	486.8		1.3117	
	2	664321	1402.0	473.8		1.2768	
	3	607574	1810.0	335.7		0.9045	
	average	699826	1637.3	432.1	83.8	1.1643	0.2257
158(A)	1	181072	740.7	244.5		0.6431	
	2	126321	352.7	358.2		0.9422	
	3	135615	484.7	279.8		0.7360	
	average	147670	526.0	294.2	58.2	0.7738	0.1531
158(4nt-3'extenion)	1	615420	1720.0	357.8		0.9641	
	2	255034	670.0	380.6		1.0257	
	3	622647	2072.0	300.5		0.8097	
	average	497701	1487.3	346.3	41.3	0.9332	0.1112
155-158(Δ4-3'deletion)	1	655442	1856.0	353.1		0.9516	
	2	980470	2248.0	436.2		1.1752	
	3	912339	2364.0	385.9		1.0399	
	average	849417	2156.0	391.7	41.8	1.0556	0.1126
144-158(Δ15-3'deletion)	1	5058874	11660.9	433.8		1.1690	
	2	6115765	14186.5	431.1		1.1616	
	3	8319979	19118.5	435.2		1.1726	
	average	6498206	14988.6	433.4	1.26	1.1677	0.0056
SL-rearrange1	1	1202130	3598.2	334.1		0.9002	
	2	1358618	4142.0	328.0		0.8838	
	3	1429717	4352.1	328.5		0.8852	
	average	1330155	4030.8	330.2	188.88	0.8897	0.0091
SL-rearrange2	1	32465.0	97.92	331.5		0.8933	
	2	35032.0	88.43	396.2		1.0675	
	3	57841.0	140.95	410.4		1.1057	
	average	41779.3	109.10	379.4	42.0	1.0222	0.1132
10nt-5'extention	1	6008790	21995.0	273.2		0.7361	
	2	4488206	16709.7	268.6		0.7237	
	3	5790027	21307.8	271.7		0.7322	

		average	5429008	20004.2	271.2	2.35	0.7307	0.0063
	20nt-5'extention	1	5398431	12406.5	435.1		1.1725	
		2	6072558	14146.4	429.3		1.1567	
		3	6551830	15573.7	420.7		1.1336	
		average	6007607	14042.2	428.4	7.26	1.1542	0.0196
	Δ34-5'deletion	1	6840269	14787.3	462.6		1.2464	
		2	6360454	15376.0	413.7		1.1146	
		3	4576775	9979.4	458.6		1.2358	
		average	5925833	13380.9	445.0	16.5	1.1989	0.0732
	35S::OsMac3-GUS	1	607739	3594.0	169.1		0.4556	
		2	540931	3848.0	140.6		0.3788	
		3	611346	3342.0	182.9		0.4929	
		average	586672	3594.7	164.2	21.597	0.4424	0.0582
	35S::GUS	1	161052	3446.0	46.7		0.1259	
		2	78972	2356.0	33.5		0.0903	
		3	80087	2140.0	37.4		0.1008	
		average	106704	2647.3	39.2	6.7901	0.1057	0.0183

Figure 4	Sample		GUS activity (Raw)	Value of Cell factor	Relative GUS activity	SD	Translational efficiency	SD
	SPK::GUS	1	369.3	270.7	1.36		0.3063	
		2	-448.7	588.7	-0.76		-0.3721	
		3	3696.3	644.7	5.73		3.0658	
		average	1205.7	501.3	2.11	3.31	1.0000	1.8209
	SPK::dMac3-GUS	1	13732.3	528.7	26.0		63.8516	
		2	14536.3	396.7	36.6		67.5899	
		3	23914.3	752.7	31.8		111.1950	
		average	17394.3	559.3	31.5	5.34	80.8788	26.3211
	UBQ::GUS	1	3849.7	2004.7	1.92		1.0842	
		2	3246.7	1728.7	1.88		0.9144	
		3	3555.7	1918.7	1.85		1.0014	
		average	3550.7	1642.7	1.88	0.03	1.0000	0.0849
	UB::dMac3-GUS	1	6990.7	1942.7	3.60		1.7998	
		2	7526.7	1832.7	4.11		1.9378	
		3	7124.7	1702.7	4.18		1.8343	
		average	7214.0	2081.3	3.96	0.32	1.8573	0.0718

Supplementary Table S1. The raw data of the GUS activity, relative GUS activity, and relative translational efficiency. Because the values varied depending on the viability of the cells used in each experiment, the relative GUS activities were adjusted based on the value of the observed GUS activity of dMac3 in a simultaneous experiment in the experiment for Figure 3. Values of cell factor are taken by the results of qRT–PCR that were multiplied by the cell factors depending on the cell viability, whose values were obtained by the GUS activity on the dMac3 reporter gene.
